# The Comparison of Stress Distribution with Different Implant Numbers and Inclination Angles In All-on-four and Conventional Methods in Maxilla: A Finite Element Analysis

**DOI:** 10.15171/joddd.2015.044

**Published:** 2015-12-30

**Authors:** Fariba Saleh Saber, Shima Ghasemi, Rodabeh Koodaryan, Amirreza Babaloo, Nader Abolfazli

**Affiliations:** ^1^Associate Professor, Department of Prosthodontics, Faculty of Dentistry, Tabriz University of Medical Sciences, Iran; ^2^Assistant Professor, Department of Prosthodontics, Faculty of Dentistry, Tabriz University of Medical Sciences, Iran; ^3^Assistant Professor, Department of Periodontics, Faculty of Dentistry, Tabriz University of Medical Sciences, Iran; ^4^Associate Professor, Department of Periodontics, Faculty of Dentistry, Tabriz University of Medical Sciences, Iran

**Keywords:** Dental implant, finite element analysis, maxilla

## Abstract

***Background and aims. ***All-on-four technique involves the use of tilted implants to allow for shorter cantilevers. This finite element analysis aimed at investigating the amount and distribution of stress in maxillary bone surrounding the implants with all-on-four vs. frequently used method with six implants technique using different numbers and inclination angles.

***Materials and methods.*** A 3D edentulous maxillary model was created and implants were virtually placed anterior to the maxillary sinus and splinted with a superstructure. In total, five models were designed. In the first to the fourth models, four implants were placed with distal implants inclined 0, 15, 30, and 45 degrees, respectively. In the fifth model, six vertical implants were placed. 100 N loading was placed in the left most distal region of the superstructure. Maximum von Mises stress values were evaluated in cancellous and cortical bone.

***Results.*** The maximum stress values recorded in cancellous and cortical bone were 7.15 MPa and 51.69 MPa, respectively (model I). The reduction in stress values in models II to V 6%, 18%, 54%, and 24% in cancellous bone and 12%, 36%,62%, and 62% in cortical bone, respectively.

***Conclusion.*** Increasing the inclination in posterior implants resulted in reduction of cantilever length and maximum stress decline in both cancellous and cortical bone. The effect of cantilever length seems to be a dominant factor which can diminish stress even with less number of implants.

## Introduction

Alveolar bone height is often lower in the posterior region than in the anterior in maxillary edentulous arches.^1^ In this regard, the proposed methods in the treatment of atrophic posterior maxilla include bone grafting alone, Lefort I osteotomy with the interpositional bone graft, sinus floor elevation, or use of zygomatic implants.^2-6^ The post-surgical complications of these treatments are donor area morbidity, loss of bone graft or implant, sinusitis, osteomyelitis, and fistula.^1^

The all-on-four concept was presented in mid 1990s as a way to treat fully edentulous and atrophic mandible with fixed prosthesis without advanced surgery. The aim of this concept was maximum use of existing bone and having early function of implants. Application of four implants instead of six or more, and avoiding advanced surgical techniques will also reduce the involved expenses. This method is especially effective in the upper jaw as the fastest bone height loss occurs in posterior maxilla, and with this technique permits longer and stronger implants placement.^7,8^ In this way, four implants are placed in an edentulous jaw. Two vertical anterior fixtures at the lateral incisors sites and two posterior long fixtures with distal angulations at the premolars sites are placed.^9^

Angulations of posterior implants will increase prosthetic support in posterior arch area but maximum angle of 45 degrees should be considered.^7;9^ Several reports of successful treatment have been published.^10-14^ The more posterior tilt of the implants is, the lower the posterior cantilever will be, which is also expected to lower the amount of stress to the implants.^5;15^ However, the angular forces to the implants are in question. Some authors believe that if the loads onto the implants are angled, peri-implant bone resorption will happen due to shear forces in the implant–bone surface.^1^

According to a clinical study with 5-year follow-up, the placing of tilted implants is an effective and safe alternative in the treatment of atrophic maxilla. The advantages of this method include the possibility of placing longer implants and increasing the implant-bone interface, omitting or reducing the length of cantilever in the prosthesis, placing implants in the patient's existing bone and avoiding complicated surgical techniques.^15^ Takehashi et al^9^ simulated all-on-four conventional methods in mandible using a finite element model. According to the results, due to the less length of cantilever in all-on-four technique, the stress around the implants is less than the stress in six vertical implants. In this simulation model, however, the mandible was designed as a homogenous block.^9^

Bevilacque et al^16^ recommended tilted implants as a substitution to the maxillary fixed prosthesis with vertical implants and posterior cantilever using a homogenous model for maxillary bone. In their study, however, the amount of stress was evaluated at the interface of bone and implant and the stress in distant bone was not measured, and no comparisons were made with the results of conventional vertical placement method.^16^ The present finite element analysis aimed at investigating the amount and the distribution of stress in maxillary bone surrounding the implants with all-on-four vs. the often employed method for vertical implant placement, using different numbers and inclination angles of implants.

## Materials and Methods

In this study, three-dimensional finite element models of maxilla, implant fixtures, and the superstructure were used to investigate the amount and distribution of stress in maxillary cortical and cancellous bone.

The 3D model of maxilla was developed from the computerized tomography (CT) data of a patient with neurological difficulties and without any craniofacial deformity. CT-scan image control system (Mimics: Materialise Interactive Medical Image Control System; Leuven, Belgium) was used to model the maxilla. Cortical bone thickness was considered to be 1 mm in the anterior area to the canines and 0.7 mm to their posterior.^17^

**Figure 1.**
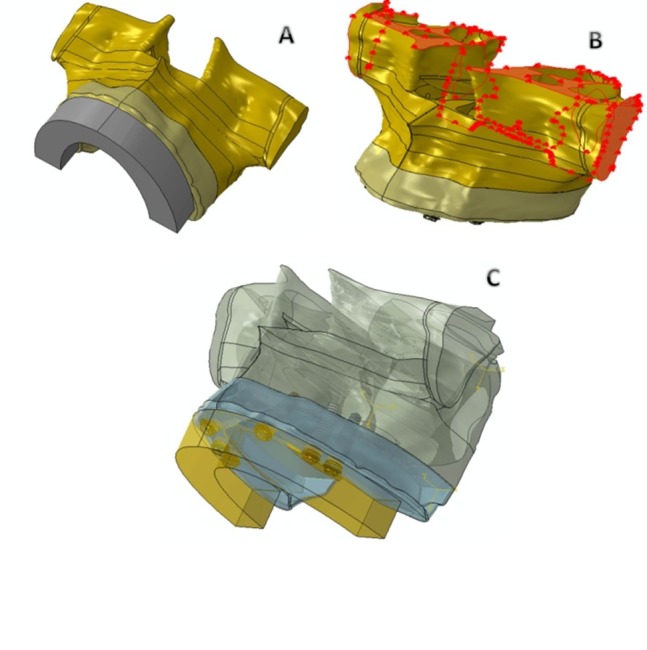
A) Superstructure model. B) Boundary conditions of designed maxillary model. C) The fifth model with six vertical implants and the load direction is featured.

Two implants (Nobel Replace; Nobel Biocare AB, Göteborg, Sweden) with a 4.3-mm diameter and 13- and 16-mm lengths and two multi-unit abutments of zero degree angle and 1-mm collar and 30 degrees angle with 3.5-mm collar height were selected. The optical digitizing system ATOS II (GOM, Braunschweig, Germany) was used to digitize the implants and abutments. The measured data was transferred to a modeling software (3D CAD 2010, SolidWorks, Concord, US) to construct the solid models. Superstructure model of 10-mm height and bucco-lingual width and periphery length of 93 mm (which includes sum of the total meiso-distal width of the right first molar to the left first molar)^18^ was designed using the same modeling software (Figure 1A).

Boundary conditions were determined according to the maxillary connection to the cranial base, by which the maxillary movement is limited (Figure 1B). The movements of nodes in these areas were completely constrained. The connection between the superstructure and the implant was considered “tie” type.

The mesh value, which indicates the number of tetrahedral elements forming the study model, was 300,000 units and the number of nodes was 7,300. The values for modulus of elasticity and Poisson’s ratio of implants, abutments and superstructure were 108,500 MPa and 0.34 (titanium alloy), respectively. Anisothropic properties for cortical and cancellous bone are given in Table 1.

**Table 1:** Properties of anisotropic bone model.

**Variable**	**Cancellous bone**	**Cortical bone**
**EX (MPa)**	1,148	12.600
**EY (MPa)**	210	12.600
**EZ (MPa)**	1,148	19.400
**GXY (MPa)**	68	4.800
**GYZ (MPa)**	68	5,700
**GXZ (MPa)**	434	5,700
**vYX**	0.010	0.300
**vZY**	0.055	0.390
**vZX**	0.322	0.390
**vXY**	0.055	0.300
**vYZ**	0.010	0.253
**vX Z**	0.322	0.253

In total, five models were designed. In all models, implants were placed anterior to the anterior wall of maxillary sinus. In the first model, four vertical implants were inserted in lateral incisor and first premolar sites bilaterally according to the protocol of Zarb et al.^19^ In the second to fourth models anterior implants were vertical and posterior implants were tilted distally 15, 30, and 45 degrees to the vertical line, respectively. In the fifth model six vertical implants (conventional method) were placed virtually in the lateral incisor as well as first and second premolar areas bilaterally (Figure 1C).^20^ All implants in all models were 13 mm in length, except posterior implants in the third and fourth models that were 16 mm because of more distal tilt. The lines drawn along apices of posterior implants in first to fourth model converged at a certain point under first premolar site in all four models. While the superstructure length was fixed in all models, the lengths of distal cantilevers were 20, 16, 12 and 2.5 mm in first to fourth models, respectively, and 13 in fifth model. Loading at a force of 100 N was placed on the left most distal region of the superstructure.^14;21^

The analysis of final five models was performed by a three dimensional FE analysis package (ABAQUS V6.9-1; Simulia Corp., Providence, US).

Data was evaluated in cortical and cancellous bone. Values of von Mises stress in the bone around the implants were evaluated in five designed samples.

## Results

Stress was evaluated in both cortical and cancellous bone models. Loading tests was performed with the implants and supra structure but for the ease of viewing stress distribution areas, they have been omitted from the figures depicting the models.

### 1. von Mises Stress Distribution in Cancellous Bone

**Figure 2.**
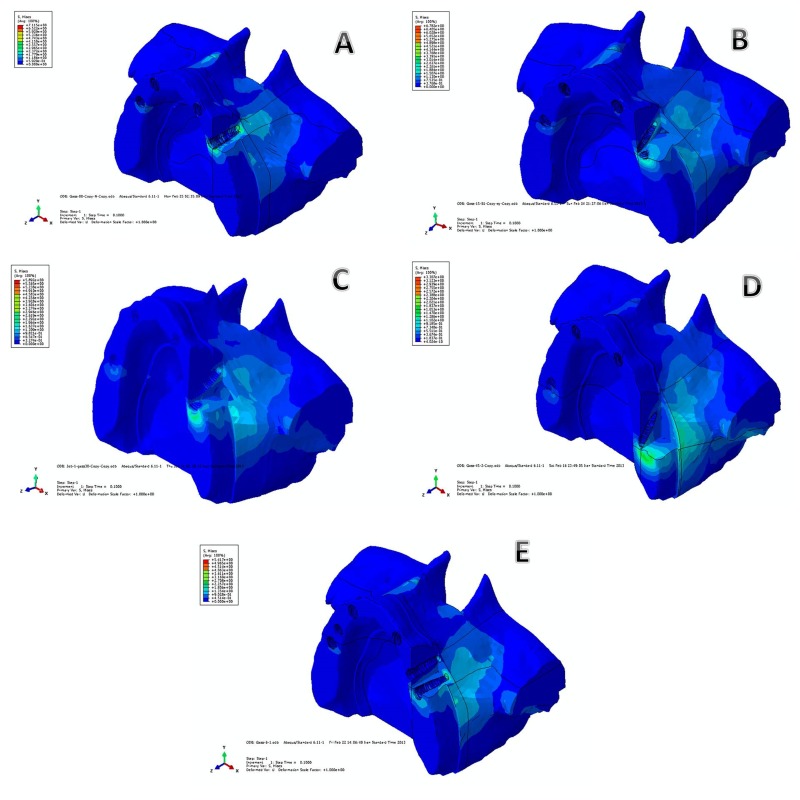
Stress distribution under loading in cancellous bone: A- model I (four implants: 0-degree anterior and posterior implants), B- model II (0-degree anterior and 15-degree posterior implants), C- model III (0-degree ante-rior and 30-degree posterior implants), D- in model IV (0-degree anterior and 45-degree posterior implants), E- in model V (six implants: 0-degree anterior and posterior implants).

A) Model I: with four vertical implants. The stress concentration in the cancellous bone around left posterior implants that is near the loading site is in two parts, in the most epical portion of bone and in the crestal region of bone around the implant. The most amount of von Mises in this model is 7.15 Mpa which is the highest amount of stress in concellous bone in all models. Stress surrounding implant is accumulated in the apical and crestal areas and have been developed less to the middle part. In apical area, stress has to some extent been developed along the implant to the distant areas from the apical end and along the anterior wall of maxilla (Figure 2A).

B) Model II: with 4 implants while posterior implants of both sides are tilted 15 degrees to distal. In this model stress of left posterior implant is, more concentrated in crestal and apical areas.

Maximum stress in this model is 6.7 MPa which has decreased 6% to the zero degree model. The difference in stress distribution to the previous model is that in the crestal area stress develops more distally and in the apical area stress is distally from the apical end of the implant (Figure 2B).

C) Model III: with 4 implants while posterior implants of both sides are titled 30 degrees to the distal. In this model, the stress in the left posterior implant is seen in two apical and crestal area but stress distribution is more in crestal than in apical. The maximum stress is 5.89 MPa which has declined 18% compared with zero model. The difference to the previous models is that, firstly, the maximum stress amount has decreased, secondly stress distribution in crestal area and along the bone crest has been more distalled and in the apical area is more distant than apex of implant and is developing to the maxilla lateral wall (Figure 2C).

D) Model IV: with 4 implants and posterior implants of both sides are tilted 45 degrees to the distal. In this model also stress in the cervical area of the left posterior implant is spread along the crest and also under the zygomatic area of maxillary bone (distant area of implant apex). The stress near to the cervical area of implant is more than stress made under zygomatic arch, the maximum stress in cancellous bone in this model is 3.3MPa which compared with first model has decreased 54% and is the least amount among four models. Compared with the previous models, stress distribution along the crest is spread more distally. Another difference is that there is more stress on the distoapical region of implant and under zygomatic arch (Figure 2D).

**Figure 3.**
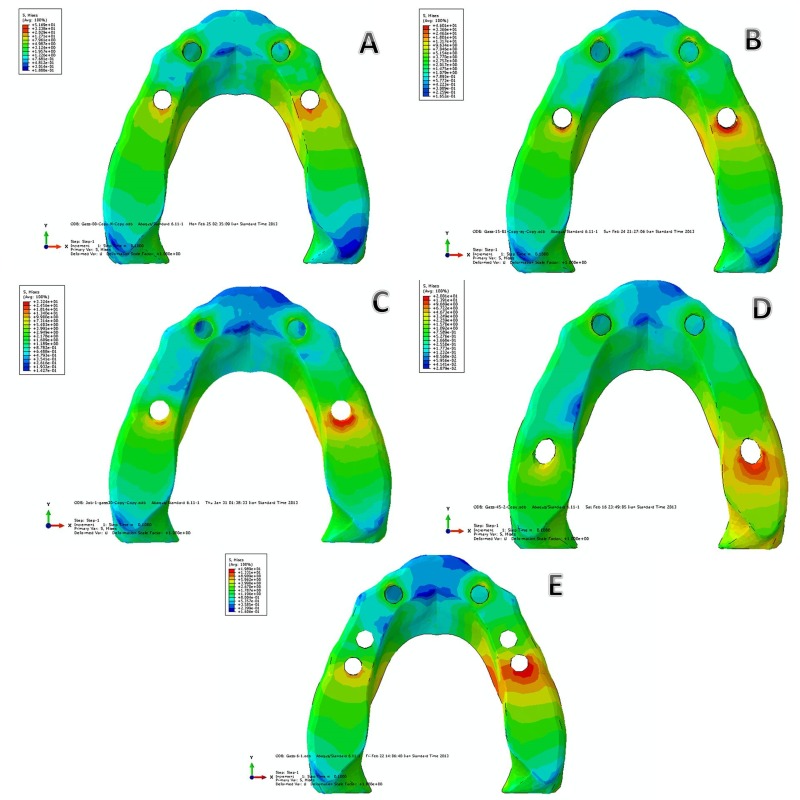
Stress distribution under loading in cortical bone: A- model I (0-degree anterior and posterior implants), B- model II (0-degree anterior and 15-degree posterior implants), C- model III (0-degree anterior and 30-degree posterior implants), D- in model IV (0-degree anterior and 45-degree posterior implants) E- in model V (six im-plants: 0-degree anterior and posterior implants).

E) Model V: with six vertical implants. Also in this model the stress concentration in the left posterior implant is in two parts, in the most apical portion of bone and in the crestal region of bone around the implant. The stress distribution is more in apical than in crestal region. The most amount of von Mises in this model is 5.41 Mpa in concellous bone which has decreased 24% compared with the first model (Figure 2E and 3).

### Comparing of General Results in Cancellous Bone

1. In the first to fourth model, around the cervical area of left side posterior implant stress levels are decreased with increasing the angle but more distributed along the crestal bone to the distal side. In other words, the more vertical posterior implant and the longer the cantilever prosthesis, the more the amount of stress and the more concentrated the distribution of them.
2. In the right posterior implant: in each of models stress is accumulated in the crestal bone. The stress rate is of the second rate after the left posterior implant.
3. Anterior implants: In all four models has minimal stress compared with other implants and almost equal to each other.

### 2. von Mises Stress Distribution in Cortical Bone

A) Model I: In the left posterior implant stress is extensive in crestal bone of the distal aspect of implant. The maximum von Mises stress is 51.69 MPa which has the highest stress in comparison to other implants of all models. Also the stress level is much more in the underlying cancellous bone (Figure 3A).

B) Model II: In left posterior implant stress is more extensive in distal region of crestal bone than in model I. The maximum von Mises stress is 46 MPa which has the most stress in comparison to other implants of this model. In comparison with model I the maximum von Mises stress level has decreased 12%. Also stress level is for more than underlying cancellous bone (Figure 3B).

C) Model III: In left posterior implant stress is accumulated in the distal area of the crestal bone and is expended more distal than the two previous models. The maximum Von Mises stress is 33.24 MPa which has the most stress in comparison to other implants of this model. In comparison to model I the maximum stress has reduced 36%. Also stress level is more than the underlying cancellous bone (Figure 3C).

D) Model IV: In left posterior implant stress is accumulated in the distal area of crestal bone and is more distally expended than all other models. The maximum von miss stress is 20 MPa which is the most stress in cortical bone of this model, also is more than the underlying cancellous bone. In comparison to zero degree, the maximum Von Mises has declined 62% then it is the least stress value in cortical bone in all five models (Figure 3-D).

**Figure 4.**
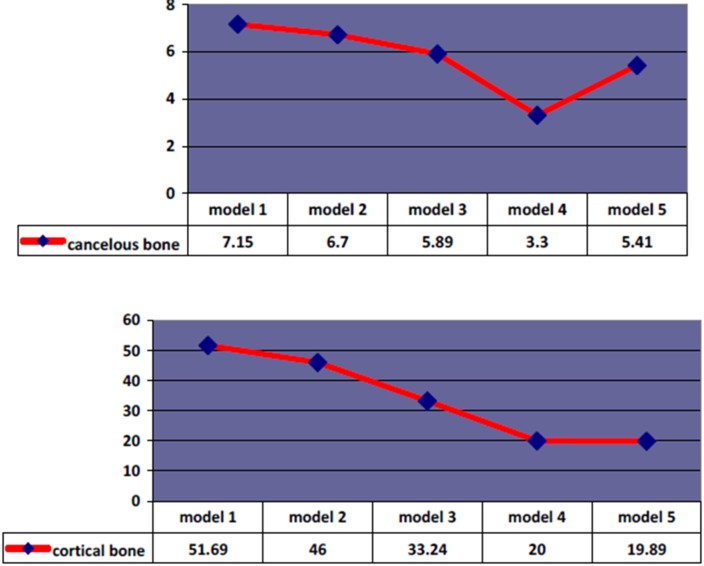
Stress values in five models: A- cancellous bone, B- cortical bones.

E) Model V: In left posterior implant stress is accumulated in the distal area of crestal bone. The maximum von miss stress is 19.89 MPa which has the most stress of this model and more than the underlying cancellous bone. In comparison to zero degree, the maximum Von Mises has declined 62% and is equivalent to model IV (Figure 3E & 4).

In each of the models:

- In the right posterior implant the stress expan-sion is in the distal area of implant.

- In anterior implants, the stress of the right and left implants are almost equal to each other and stress levels are lower compared with posterior implants.

## Discussion

In this study, we attempted to investigate the effect of number and inclination angle of implants on the amount and distribution of stress in maxilla in all-on-four and frequently used methods simulated by finite element analysis. Modifications in the approach used in previous articles were introduced in order to maximize the simulation between finite element models and the clinical situation. We used shorter implants that those usually employed in the clinical situation. The implants system selected was Nobel Biocare which offers special abutments for all-on-four technique. Another difference between this study and the study of Bevilacque was the simultaneous investigation of stress distribution in this study compared with evaluation of separate models of cancellous and cortical bone in the previous study, while in fact these two bones are not isolated ones. Our study is based on a model composed of natural morphology and anatomy of both bones, as the cortical bone thickness is 1 mm in anterior of canines and 0.7 mm in posterior areas.^17^ Another difference with previous studies was the anisotropic properties of the bone model in the current study, which is closer to reality, compared with the isotropic design of the bone model in previous studies.^9,16^

The results showed that stress declined around the cervical area of posterior implants in cancellous and cortical bone as the angle increased and spread distally along crestal bone. In other words, the more vertical are the posterior implants and the longer the cantilever prosthesis, the higher and concentrated becomes the von Mises stress.

In all models, the amount of stress in cortical bone is far more than the underlying cancellous bone similar to results of other studies. This finding is in line with previous studies.^16,22-23^ This may, in fact, happen because of the higher modulus elasticity of cortical bone which causes more stress. In both cancellous and cortical types of bone, the maximum stress is around posterior implants on the side of loading. By increasing the angle of posterior implants in first to fourth model, stress decreased so that the minimum was seen in the fourth model (45 degrees). This can be because of shortening the cantilever and is consistent with previous studies.^9,16^ Comparing the fourth model (with four implants) and fifth model (with six vertical implants), the amount of stress in cortical bone was approximately equal but was lower in cancellous bone of the fourth model. This shows that applying two more implants but with higher cantilever lengths did not decreased the stress in cancellous bone. Therefore, it might be concluded that the effect of cantilever length is the primary factor and can diminish stress even with less numbers of implants. Indeed, this study shows that the decrease in the cantilever length with four implants (fourth model) significantly decreased the amount of stress, compared with longer cantilevers with six implants (fifth model) in cancellous bone in maxilla. However, this decrease was not significant in cortical bone. This was in approximate consistence with the findings of Takehashi et al.^9^

## Conclusions

1) By increasing the angle of posterior implants, the stress declined in both cancellous and cortical bones but the reduction is only significant in cancellous bone.

2) By increasing angle of posterior implants, stress spread more distally.

3) The effect of cantilever length is a dominant factor and can diminish stress even with lower number of implants.

## Acknowledgments

This research was carried out by financial support of the Vice Chancellor for Research at Tabriz University of Medical Sciences. The authors declare that they have no competing interests.
